# Evaluation of Intracranial Pressure in Patients with Severe Brain Injury Using Contrast-Enhanced Ultrasound: A Pilot Study with Preliminary Findings

**DOI:** 10.1007/s12028-026-02447-w

**Published:** 2026-02-10

**Authors:** Xixi Sun, Jiayuan Chai, Qian Li, Lizhi Zheng, Jia Sun, Nan Cao, Caibao Hu, Bin Huang

**Affiliations:** 1 Department of Ultrasound, Zhejiang Hospital, No. 1229 Gudun Road, Xihu District, Hangzhou, 310013 Zhejiang Province China; 2 The Second Clinical Medical College, Zhejiang Chinese Medical University, Hangzhou, 310053 Zhejiang Province China; 3 Department of Ultrasound, The First People’s Hospital of Pinghu, No. 500 Sangang Road, Danghu Street, Pinghu, 314200 Zhejiang Province China; 4 Department of Critical Care Medicine, Zhejiang Hospital, No. 1229 Gudun Road, Xihu District, Hangzhou, 310013 Zhejiang Province China

**Keywords:** Contrast-enhanced ultrasound, Quantitative analysis, Severe brain injury, Intracranial pressure, Central retinal artery

## Abstract

**Background:**

Contrast-enhanced ultrasound (CEUS) has great potential for assessing increased intracranial pressure (ICP); however, the most appropriate parameters remain unknown. This study aimed to explore the application of quantitative analysis of CEUS diagnosing increased ICP in patients with severe brain injury after surgery, and to provide a novel idea for the non-invasive evaluation of ICP.

**Methods:**

This observational study included 34 patients with craniocerebral injuries admitted to the intensive care unit from May 2022 to December 2023. Patients were divided into a normal cranial pressure group (< 20 mm Hg) and an intracranial hypertension group (≥ 20 mm Hg) on the basis of the invasive ICP monitoring values. All patients underwent CEUS examination within 24 h postoperatively. Time-intensity curves of the central retinal artery and short posterior ciliary artery were generated using CEUS quantitative analysis software. Quantitative parameters were obtained, and the difference was calculated. The diagnostic accuracy of each parameter was assessed by computing the area under the receiver operating characteristic curve (AUC).

**Results:**

In total, 15 patients (44%) had intracranial hypertension. The arrival time difference (ΔAT) and peak time difference (ΔTP) were significantly higher in the intracranial hypertension group than in the normal cranial pressure group (*P* < 0.001 and *P* = 0.010, respectively). The peak intensity difference (ΔPkI) was significantly lower in the intracranial hypertension group than in the normal cranial pressure group (*P* = 0.025). The diagnostic accuracy of ΔAT for identifying intracranial hypertension was excellent, with an AUC of 0.944 [95% confidence interval (CI) 0.874–1.014], which yielded an optimal cutoff value of 0.86 s with 93.3% sensitivity (95% CI 0.849–1.017) and 84.2% specificity (95% CI 0.719–0.964). The AUC of ΔAT was significantly higher than that of ΔTP and ΔPkI (AUC 0.761 and 0.721, respectively; both *P* < 0.05).

**Conclusions:**

The CEUS quantitative analysis parameter, ΔAT, is a promising parameter for evaluating postoperative ICP elevation in patients with severe brain injury.

## Introduction

Severe brain injury includes severe traumatic brain injury, hemorrhagic and ischemic stroke, and hypoxic-ischemic brain injury [1]. Survivors experience adverse outcomes, such as impaired cognitive function, speech disorders, and motor dysfunction, which place a heavy burden on the patient’s family, health system, and socioeconomic status [2, 3]. Notably, the poor prognosis in patients with brain injury is closely related to cranial hypertension. Both computed tomography (CT) and magnetic resonance imaging (MRI) can be used for noninvasive screening of cranial hypertension [4, 5]. However, patients with brain injuries have difficulty moving, and there is a risk of deterioration. Therefore, CT/MRI is not the best choice. Increasing attention has been paid to the application of ultrasound in the noninvasive evaluation of increased intracranial pressure (ICP) [6, 7]. The use of ultrasonographically measured optic nerve sheath diameter (ONSD) and transcranial Doppler (TCD) to monitor cerebral hemodynamics for predicting elevated ICP has been extensively reported [8–11]. However, in some patients with severe brain injury, the ONSD remains dilated with a certain degree of lag, even after the ICP has decreased to within the normal range [12]. The accuracy of TCD examination and measurement is affected by many factors and has limitations. Contrast-enhanced ultrasound (CEUS) has the ability to track blood perfusion in tissue and lesion sites in real time, which in turn provides information about microvascular hemodynamics [13, 14]. For a more precise analysis, CEUS quantitative perfusion analysis was applied to visually evaluate the perfusion of the contrast agent over time in the target area [15, 16], aiming to provide more detailed microvascular quantitative data to assist clinical diagnosis and treatment.

The central retinal artery (CRA) is the primary branch of the ophthalmic artery. The CRA is wrapped by the optic nerve sheath and is subjected to the combined effects of intrathecal cerebrospinal fluid pressure and intravascular pressure [17], suggesting that the CRA may be a new direction for assessing increased ICP. Further anatomical investigations revealed that the short posterior ciliary artery (SPCA), located on both sides of the optic nerve, is a secondary branch of the ophthalmic artery [18], which is close to the CRA and located at the outer edge of the optic nerve sheath. Consequently, it could be visualized simultaneously with the CRA on the same ultrasonic plane. Moreover, the hemodynamic changes were less susceptible to the influence of cerebrospinal fluid pressure within the sheath. Therefore, this study intended to investigate the performance of CEUS quantitative analysis of postbulbar microvascular perfusion for diagnosing the ICP elevation in patients with severe brain injury.

## Methods

### Study Design and Participants

**Fig. 1:**
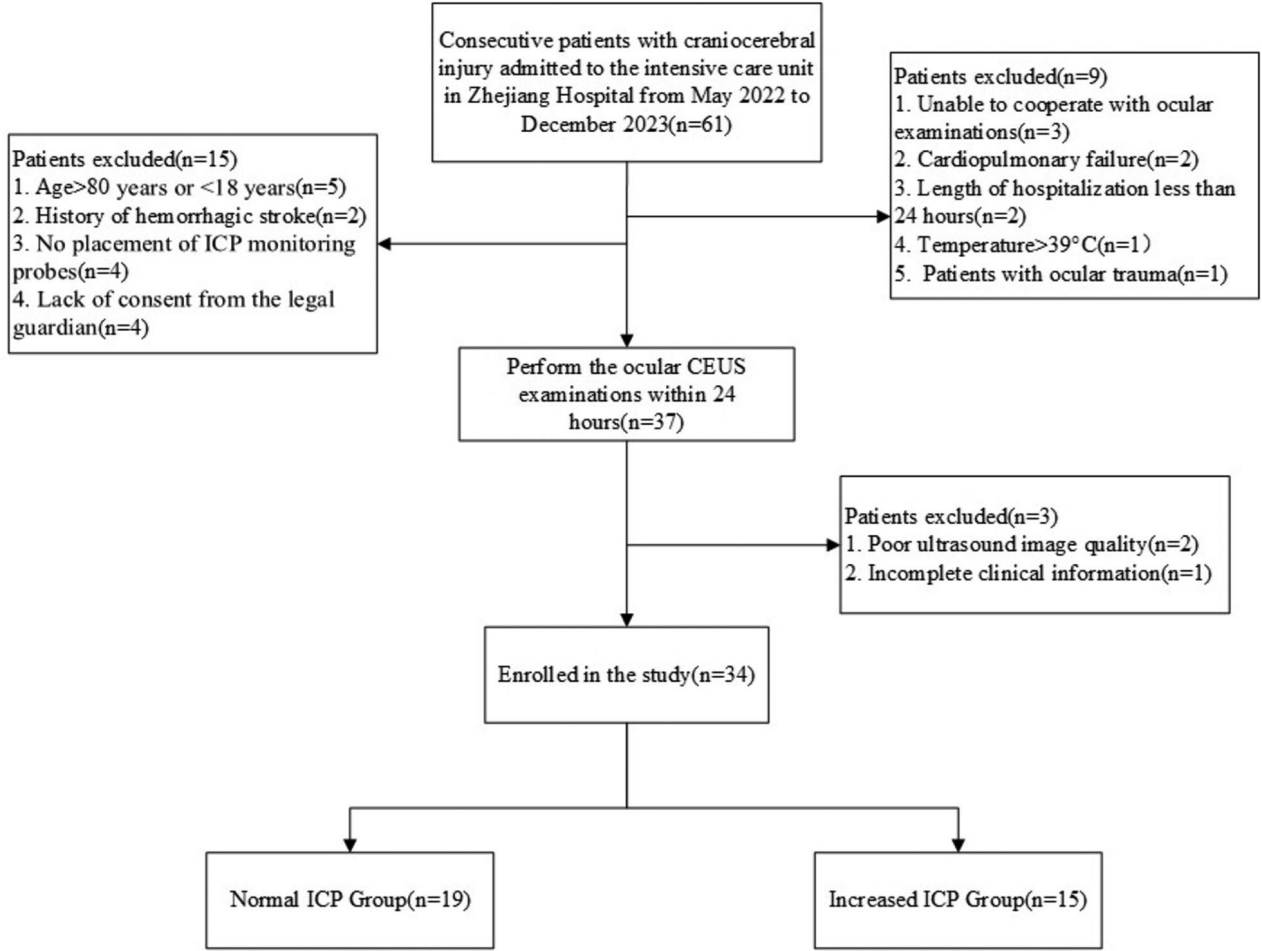
Flowchart of participants in this study

This was a retrospective case–control study conducted in the intensive care unit (ICU) of Zhejiang Hospital from May 2022 to December 2023. This study was approved by the Medical Ethics Committee of Zhejiang Hospital (approval no. 2021-139K). All patients’ families were informed about the trial before enrollment, and they signed an informed consent form. The studied subjects were adult patients with severe brain injury. All patients underwent invasive ICP monitoring (via intraventricular, intraparenchymal, or subdural routes) after admission, with continuous ICP monitoring maintained for 24 h postoperatively. The inclusion criteria included: (1) age ≥ 18 years and ≤ 80 years; (2) initial diagnosis of traumatic brain injury or hemorrhagic stroke; (3) patients who underwent surgical treatment (cerebral hematoma removal with or without decompressive craniectomy and ventricular drilling with ventricular drainage) and placement of ICP monitoring probes; and (4) signed informed consent for CEUS examination. The exclusion criteria included: (1) patients with ocular trauma, skin infection, and history of ocular surgery, glaucoma, optic neuritis, optic nerve tumor, diabetic retinopathy, and other ophthalmic diseases; (2) patients with a history of serious vascular lesions (ocular artery stenosis, occlusion of the CRA); (3) patients under medications that affect the cerebrospinal fluid; (4) patients with cardiopulmonary failure; (5) patients who were unable to cooperate with ocular examinations; (6) patients with high fever; (7) patients with a history of allergy to ultrasonic contrast agents; (8) patients who were treated in the intensive care unit for less than 24 h; or (9) patients who had lost data or had poor quality of the acquired images. The process of patient screening is shown in Fig. 1.

### Main Equipment

The ultrasound instruments used were the LOGIQ E11 color Doppler ultrasound diagnostic apparatus (General Electric Company, USA) and M9 portable color Doppler ultrasound diagnostic apparatus (Shenzhen Mindray Biological Co., Ltd.). The probes were 3–12 or 3–14 MHz high-frequency linear array probes. The ultrasound contrast agent used was sulfur hexafluoride micro contrast agent (SonoVue, Bracco Company, Italy).

### CEUS

**Fig. 2:**
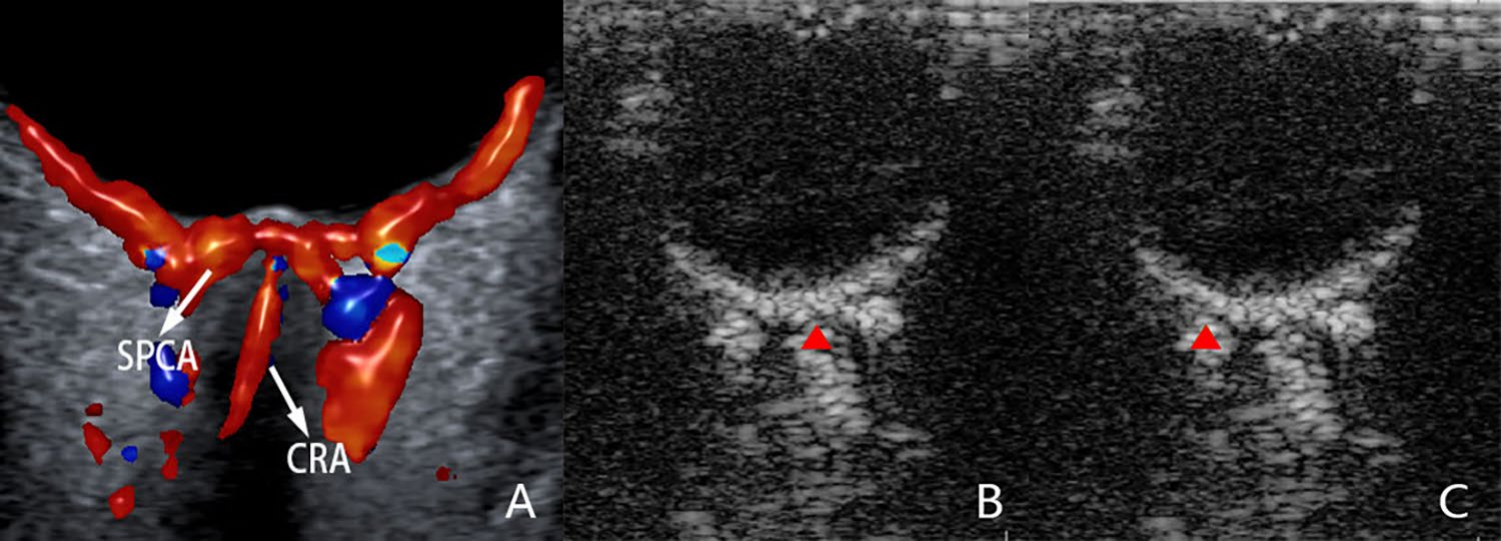
**A** The position of CRA and SPCA behind the eyeball in color Doppler mode (white arrow). **B** The triangle is the region of interest selected for CEUS quantitative analysis of the CRA. **C** The triangle is the region of interest selected for CEUS quantitative analysis of the SPCA. Abbreviations: *CEUS* contrast-enhanced ultrasound, *CRA* central retinal artery, *SPCA* short posterior ciliary artery

*CEUS image acquisition*: CEUS measurements were performed within 24 h after surgery, requiring patients to be in a stable respiratory and calm state. The patient was placed in the supine position with the eyes closed and head elevated by 20°–30°. Simultaneously, the skin around the eyelids was cleaned, and transparent eye patches were used to cover the patient’s eyelids. The CEUS mode was selected, and the mechanical index should be lower than 0.1, basically set at approximately 0.07 [19]. The image gain was set for automatic optimization. Image depth was adjusted to approximately 4 cm behind the eye. The probe was gently placed on the eyelid along the horizontal section and the angle of the probe was finetuned until a well-defined hyperechoic strip-like structure with a clear edge was observed behind the eyeball, which was the optic nerve sheath. Subsequently, after confirming the position of CRA and SPCA using color Doppler mode, as shown in Fig. 2, 2.2 ml of SonoVue ultrasound contrast suspension was withdrawn using a syringe and injected into the patient’s previously established venous access (elbow vein or subclavian vein) through the bolus injection. The tube was quickly flushed with 5.0 ml normal saline, and the contrast agent was injected. Simultaneously, the time and dynamic storage keys were pressed to observe the perfusion of the CRA and SPCA contrast agent microbubbles for more than 1 min. CEUS was performed in the transverse plane of the right eye of each patient without any special circumstances. The left eye was considered under special circumstances, such as poor image quality of the right eye. If the ICP in the intracranial hypertension group increased during the CEUS examination, we will immediately report this situation to the clinician and let the clinician decide whether to take measures to reduce the ICP. The image data from the entire imaging process were stored on the hard disk of the instrument for subsequent analysis.

*CEUS quantitative analysis*: Video data of the retrobulbar microvessels stored on the hard disk of the instrument in the early stages were retrieved for offline analysis. Contrast analysis software, Qontrast 4.0, was used to draw a triangular zone of interest with a side length of approximately 2 mm at the location where the CRA display was more obvious on the CEUS image—approximately 3 mm from the posterior wall of the eyeball—as shown in Fig. 2. The time-intensity curve (TIC) of the CRA was automatically drawn using analysis software, and the arrival time (AT), peak time (TP), mean transit time (MTT), and peak intensity (PkI) of the blood flow were calculated. Similarly, a triangular region of interest with a side length of 2 mm was drawn at the SPCA imaging position at the same level as the CRA as a control, as shown in Fig. 2. The TIC curve of the SPCA was automatically drawn using analysis software, and the AT, TP, MTT, and PkI values ​​of the blood flow were recorded. Finally, the results of the TIC curves of the two arteries were used to calculate ΔAT = AT_CRA_ – AT_SPCA_, ΔTP = TP_CRA_ – TP_SPCA_, ΔMTT = MTT_CRA_ – MTT_SPCA_, and ΔPkI = PkI_CRA_ – PkI_SPCA_.

### Data Collection

All data collection was performed by two ultrasonographers. CEUS was performed by an ultrasound physician blinded to the invasive ICP with rich experience in CEUS examination. Meanwhile, another ultrasonographer is responsible for recording the patient’s relevant clinical data, including sex and age, and mean arterial pressure (MAP), heart rate, body temperature, arterial oxygen partial pressure (PaO_2_), arterial carbon dioxide partial pressure (PaCO_2_), invasive ICP monitoring value, and Glasgow coma score. The patient’s blood pressure, heart rate and body temperature were read and recorded from the ECG monitor. PaO_2_ and PaCO_2_ were obtained from arterial blood gas analysis results at 12 h after surgery. Invasive ICP monitoring values were recorded while the CEUS was being performed. The time of CEUS examination of each patient was controlled within 2 min. The ICP we recorded is a dynamic change interval within these 2 min. The Glasgow coma scale score was assessed by the clinicians upon admission. ICP monitoring and management were conducted following a protocol-driven approach that encompassed sedation, optimized cerebral perfusion pressure, administration of hyperosmolar fluids, and induction of hypothermia in line with institutional guidelines [MAP = (systolic blood pressure + 2 × diastolic blood pressure) / 3.]

### Statistical Analysis

Statistical software SPSS 29.0 was used to analyze the data. The normally distributed data was presented as mean ± standard deviation (SD), and the data with skewed distribution was reported as median with interquartile range (IQR). The intergroup comparisons were analyzed using Student’s *t*-tests or the Mann–Whitney *U* test, as appropriate. Count data were expressed as frequency (percentage) and were compared using the *χ*^*2*^ test. The diagnostic accuracy of CEUS parameters for identifying intracranial hypertension was assessed by computing the area under the receiver operating characteristic curve (ROC) (AUC). The cutoff value was determined by identifying the maximum of the Youden index. The AUC of each CEUS parameter was compared using a nonparametric approach proposed by DeLong et al. [20]. *P* < 0.05 was considered statistically significant.

## Results

### Baseline Patient Characteristics

A total of 61 patients with craniocerebral injury were screened, and 34 patients met the inclusion criteria and were included in this study. Of the 34 included patients, 15 had intracranial hypertension. The study selection flowchart is shown in Fig. 1. There were 12 cases of hemorrhagic stroke and 7 of traumatic brain injury in the normal cranial pressure group, and 9 cases of hemorrhagic stroke and 6 of traumatic brain injury in the intracranial hypertension group. According to Table 1, the baseline characteristics were comparable between the two groups.

**Table 1:** Demographic and clinical characteristics of the two groups of patients with brain injury

Characteristic	Normal ICP group (*n* = 19)	Increased ICP group (*n* = 15)	*P* value
Man (%)	13 (68.4%)	11 (73.3%)	0.755
Age (year)	61.26 ± 11.42	58.07 ± 18.59	0.541
MAP (mm Hg)	94 ± 12	93 ± 14	0.779
PaCO_2_ (mm Hg)	38.04 ± 5.29	34.78 ± 5.37	0.086
PaO_2_ (mm Hg)	124.40 ± 36.07	115.46 ± 30.62	0.449
Heart rate (beat/min)	85.47 ± 9.95	90.13 ± 12.67	0.238
Body temperature (°C)	37.20 ± 0.44	37.49 ± 0.53	0.089
Hemoglobin (g/L)	109 ± 18	98 ± 17	0.079
GCS (score)	7.95 ± 2.39	5.73 ± 2.15	0.009

Abbreviations: *GCS* Glasgow coma scale, *MAP* mean arterial pressure, *PaO*_*2*_ partial pressure of oxygen in arterial blood, *PaCO*_*2*_ partial pressure of carbon dioxide in arterial blood

### Comparison of CEUS Parameters Between the Two Groups

**Fig. 3:**
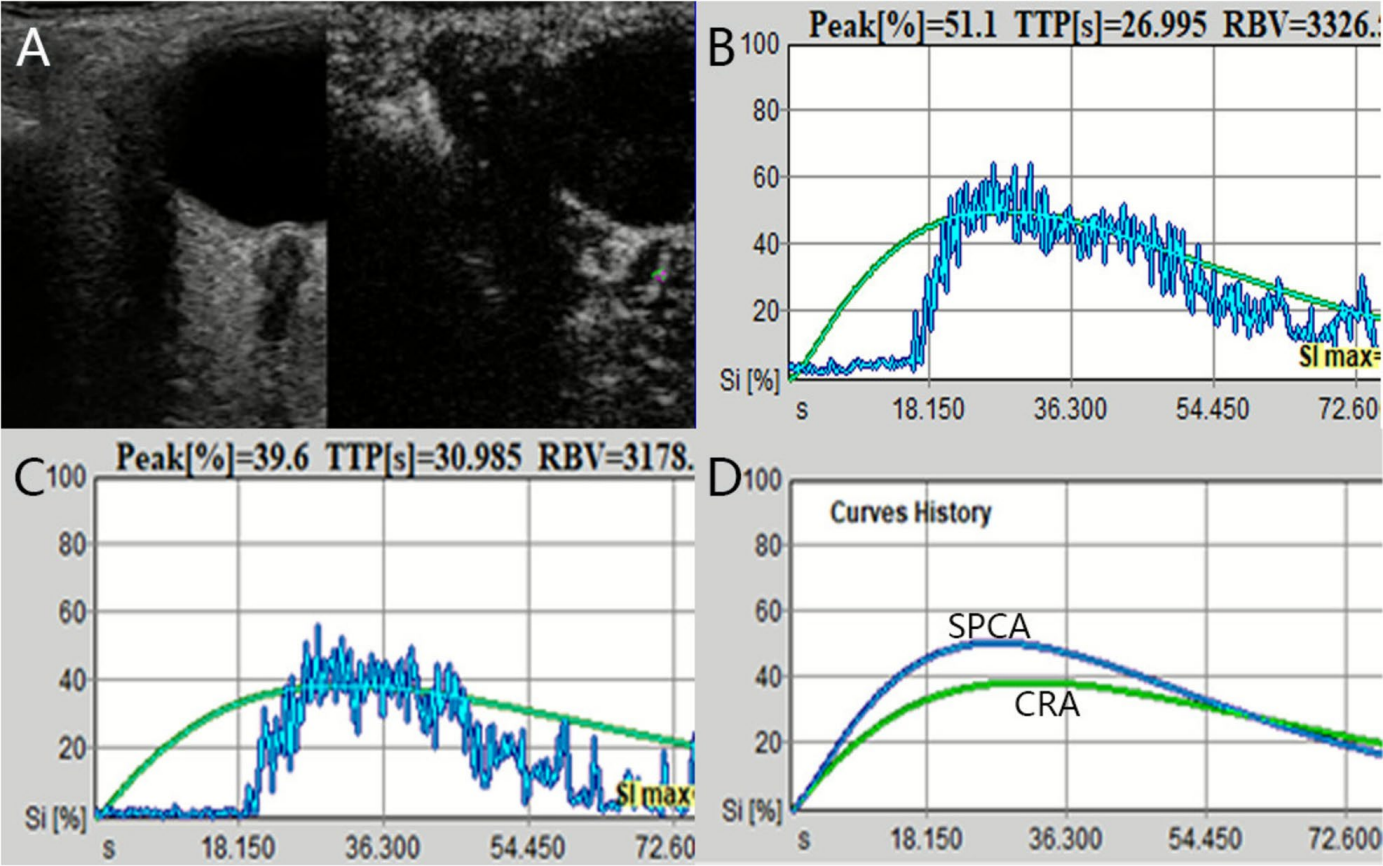
**A** CEUS image of a patient with severe intracranial hypertension. **B** The quantitative time-intensity curve analysis of SPCA, with the blue curve representing the actual SPCA measurement and the green curve showing its fitting curve. **C** The quantitative time-intensity curve analysis of CRA, with the blue curve representing the actual CRA measurement and the green curve showing its fitting curve. **D** The blue curve depicts the fitted SPCA analysis while the green curve represents the fitted CRA analysis. Abbreviations: *CEUS* contrast-enhanced ultrasound, *CRA* central retinal artery, *SPCA* short posterior ciliary artery

In this study, CEUS was performed on the right eye in all 34 patients. One patient in the elevated ICP group and one in the normal ICP group additionally underwent left eye CEUS due to poor image quality of the right eye. By observing the CEUS quantitative parameters between the two groups, it was found that in patients with brain injury whose ICP increased after the operation, the time at which CRA ultrasound microbubbles began to enhance was mostly later than that at which SPCA ultrasound microbubbles began to enhance. This phenomenon was obvious in patients whose postoperative ICP was > 40 mm Hg, as shown in Fig. 3. This could be clearly distinguished by observing CEUS images with the naked eye. Among the patients with brain injury whose ICP returned to normal after surgery, there were ten patients whose ΔAT between CRA and SPCA was within 0.5 s. When CEUS images were observed with the naked eye, the two arteries were considered to be enhanced simultaneously. In the quantitative analysis parameters of CEUS in this study, the ΔAT value of the intracranial hypertension group was significantly higher than that of the normal intracranial pressure group (*Z* = − 4.388, *P* < 0.001). The ΔTP value of the intracranial hypertension group was also significantly higher than that of the normal intracranial pressure group (*Z* = − 2.584, *P* < 0.05). Conversely, the ΔPkI value in the intracranial hypertension group was significantly lower than that in the normal intracranial pressure group (*t* = 2.356, *P* < 0.05). In addition, there was no statistical difference in ΔMTT, PkI, and MTT of CRA between the intracranial hypertension group and normal intracranial pressure group (*P* = 0.259, *P* = 0.367, *P* = 0.089, respectively, all *P* > 0.05). A comparison of two-dimensional and CEUS quantitative analysis parameters between the normal intracranial pressure and intracranial hypertension groups is presented in Table 2.

**Table 2:** Comparison of CEUS quantitative analysis parameters between the two groups

Parameter	Normal ICP group	Increased ICP group	*t*/*Z*-value	*P* value
PkI (dB)	46.600 (43.300, 49.700)	42.300 (41.000, 48.700)	− 0.902	0.367
MTT (S)	28.90 ± 7.67	36.41 ± 14.78	− 1.788	0.089
ΔAT (S)	0.344 (− 0.080, 0.764)	1.262 (0.912, 2.410)	− 4.388	< 0.001
ΔTP (S)	− 0.424 (− 1.670, 0.243)	0.811 (− 0.498, 4.803)	− 2.584	0.010
ΔPkI (dB)	0.674 ± 3.425	− 2.060 ± 3.272	2.356	0.025
ΔMTT (S)	− 3.268 ± 6.354	− 0.616 ± 7.079	− 1.150	0.259

Abbreviations: *AT* arrival time, *MTT* mean transit time, *PkI* peak intensity, *TP* peak time, ΔAT = AT_CRA_ – AT_SPCA_, ΔTP = TP_CRA_ – TP_SPCA_, ΔPkI = PkI_CRA_ – PkI_SPCA_, ΔMTT = MTT_CRA_ – MTT_SPCA_

### Results of ROC Curve Analysis for Ultrasonic Parameters

**Fig. 4:**
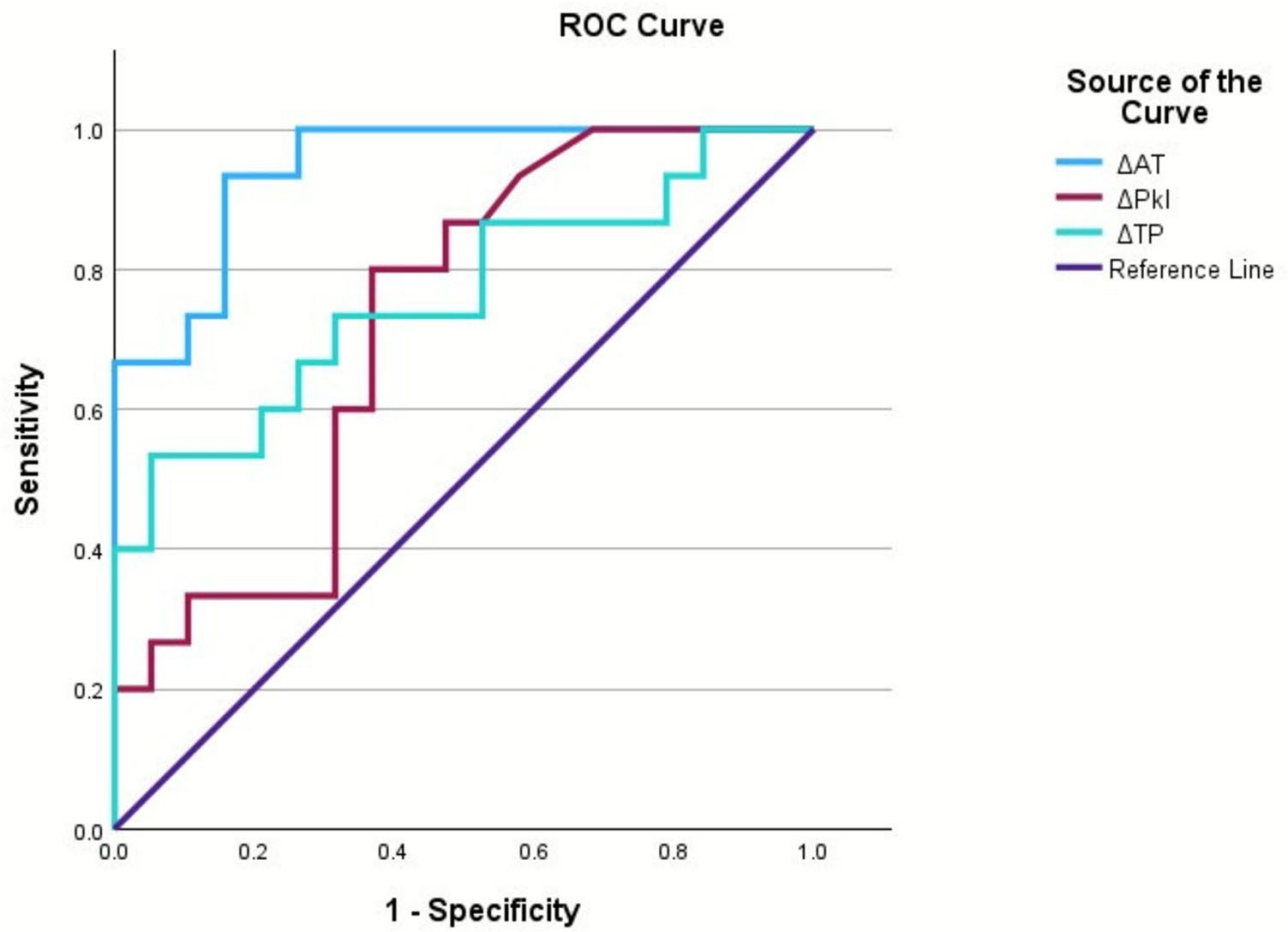
ROC curves of CEUS quantitative analysis parameters in patients with brain injury. Abbreviations: *CEUS* contrast-enhanced ultrasound, *ROC* receiver operating characteristic

The ROC curves of the CEUS quantitative analysis parameters that predicted increased ICP are shown in Fig. 4, and the results of the analysis are presented in Table 3. The diagnostic accuracy of ΔAT for identifying intracranial hypertension was excellent, with an AUC of 0.944, yielding optimal cutoff value of 0.86 s, sensitivity of 93.3%, and specificity of 84.2%. Furthermore, the AUC of ΔAT was significantly higher than that of ΔTP and ΔPkI (*P* = 0.0392,* P* = 0.0086, both *P* < 0.05).

**Table 3:** Results of ROC curve analysis for CEUS parameters

Parameter	AUC	Cutoff value	Sensitivity	Specificity
ΔAT (S)	0.944 (0.874–1.014)	0.86	93.3% (0.849–1.017)	84.2% (0.719–0.964)
ΔTP (S)	0.761 (0.593–0.930)	0.71	53.3% (0.365–0.701)	94.7% (0.872–1.022)
ΔPkI (dB)	0.721 (0.549–0.893)	0.55	63.2% (0.470–0.794)	80.0% (0.666–0.934)

Abbreviations: *AT* arrival time, *TP* peak time, *PkI* peak intensity, ΔAT = AT_CRA_ – AT_SPCA_, ΔTP = TP_CRA_ – TP_SPCA_, ΔPkI = PkI_CRA_ – PkI_SPCA_

## Discussion

ICP is an important goal-oriented parameter in the clinical treatment of patients with severe brain injury and is closely related to patient prognosis. Therefore, identifying elevated ICP levels in patients with brain injury is crucial. In this study, we found that ΔAT, ΔTP, and ΔPkI are meaningful CEUS quantitative analysis parameters for evaluating intracranial hypertension. Further ROC curve analysis showed that the area under the curve of ΔAT was the largest, and found the optimal cutoff value for diagnosing postoperative intracranial hypertension in patients with brain injury. The difference of 0.86 s is difficult to distinguish by visual observation of CEUS images. Few patients still have elevated ICP levels after craniosurgery. Most patients had an ICP that returned to the normal range after surgery. In this study, more than half of the patients in the intracranial hypertension group had an ICP between 20 and 25 mm Hg, which may lead to a smaller cutoff value of ΔAT for diagnosing postoperative intracranial hypertension. We also observed that the ΔAT was more obvious in a very small number of patients with severe intracranial hypertension (ICP > 40 mm Hg), which could be distinguished by observing CEUS images with the naked eye without TIC curve analysis clearly. Our findings suggest that ΔAT may be valuable for assessing increased ICP in situations where invasive ICP measurements are either unavailable or impractical.

Recently, the application of CEUS quantitative analysis for the diagnosis of ocular diseases has received widespread attention [21, 22]. CEUS quantitative analysis has broad application prospects in the evaluation of retroocular microvascular blood perfusion. However, few studies have been conducted on the use of CEUS for the quantitative analysis of ocular vessels in the diagnosis of brain diseases. CEUS can accurately display blood perfusion in internal and surrounding structures of the optic nerve sheath accurately [23]. Microbubbles of the contrast agent enter the CRA and SPCA, resulting in enhanced imaging. The subarachnoid space is filled with cerebrospinal fluid and imaging shows no enhancement. Under normal circumstances, both the CRA and SPCA are in a state of pressure balance. Because both arteries are branches of the ophthalmic artery and are close to each other, the contrast agent microbubbles from the CRA and SPCA arrive almost at the same time theoretically. When ICP increases, more cerebrospinal fluid enters the optic nerve sheath, widening the optic nerve sheath and increasing the internal pressure. The CRA is located inside the optic nerve and is affected by cerebrospinal fluid pressure, whereas the SPCA is located outside the optic nerve sheath and is less susceptible to cerebrospinal fluid pressure. Due to the increased ICP, the external pressure on the CRA exceeds the internal pressure, resulting in a significant increase in the blood flow resistance of the CRA. However, the SPCA is not directly compressed by the high pressure within the optic nerve sheath, and its blood flow resistance does not change significantly. As a result, the arrival time of the CRA contrast agent microbubbles may be later than that of the SPCA. Using the enhancement pattern of CEUS for description, when ICP is normal, CRA is almost “iso-advancing” relative to SPCA, whereas when ICP is elevated, CRA is “slow-advancing” relative to SPCA. This finding is consistent with our preliminary results. Our study found that the ΔAT and ΔTP values of CEUS quantitative analysis parameters were significantly higher in the intracranial hypertension group than that in the normal cranial pressure group, indicating that the arrival and peak times of the contrast agent at the CRA in the intracranial hypertension group were significantly later than those in the normal cranial pressure group.

We also found that the ΔPkI values in the intracranial hypertension group were significantly lower than those in the normal cranial pressure group, implying that the peak intensity of contrast microbubbles at the CRA decreased when the ICP increased, indicating that the overall perfusion of blood flow at the CRA decreased. Elevated intracranial pressure causes brain tissue compression, increased cerebrovascular resistance, and vascular deformation‌, which restrict blood flow into cerebral tissues [24], ultimately leading to reduced cerebral blood flow and decreased cerebral perfusion pressure‌.

In this study, more than half of the patients received contrast agent injection via the subclavian venous route. The time when CRA ultrasound microbubbles began to enhance in patients who received a bolus injection of the contrast agent through the subclavian vein was generally earlier than that in patients who received a bolus injection through the elbow vein. This is consistent with the anatomical position of the subclavian vein being closer to the eyeball and the shorter distance traveled by the contrast agent microbubbles. Considering the nonuniform approach of intravenous bolus injection of contrast agents in patients with brain injury and the differences in the speed of contrast agent bolus injection and saline flushing by each nurse, there are certain errors in the AT and TP of ultrasound microbubbles. Therefore, we did not analyze the CEUS quantitative analysis parameters AT and TP of the two groups of patients directly; we chose to compare the ΔAT and ΔTP of CRA and SPCA in the two groups. CEUS is convenient, rapid, and reproducible. When other methods for assessing ICP are not feasible, the assessment of ICP based on the quantitative analysis of CEUS is quite appealing. This provides a new diagnostic idea and approach for clinical application, further guiding precise clinical treatment.

Our study has several limitations that warrant consideration. First, the small sample size may have resulted in an overestimation of the diagnostic efficacy of CEUS parameters. The findings of this study should be further verified by expanding the sample size in the future to improve the reliability of the research. Second, we did not collect CEUS data before surgical treatment and the data from patients with intracranial hypertension after the ICP decreased to normal for comparison. This study relied solely on single-timepoint measurements without dynamic assessments, potentially failing to capture the true relationship between intracranial pressure and CEUS parameters. In future research, multiple measurements should be conducted on the same patient for longitudinal comparison to dynamically assess the relationship between intracranial pressure and various ultrasound parameters. In addition, in this study, different machine models for ocular ultrasound examination and different locations of invasive ICP monitors (intraventricular, intraparenchymal, or subdural) may cause certain deviations in the statistical analysis of data. Future studies should consider employing consistency evaluation methods to validate the impact of different ultrasound device models and invasive ICP monitor placement locations on measurement results, thereby ensuring the reliability and comparability of research findings.

## Conclusions

This pilot study suggests that the ΔAT measured by CEUS quantitative analysis is a promising parameter for evaluating postoperative ICP elevation in patients with severe brain injury. The presented study is able to develop further noninvasive ICP monitoring. Further research with an expanded sample size is needed to validate the findings of this study.
